# Associations between health-related quality of life and measures of adiposity among Filipino adults

**DOI:** 10.1371/journal.pone.0275798

**Published:** 2022-10-26

**Authors:** Joseph Capuno, Aleli Kraft, Kayleen Gene Calicdan, Owen O’Donnell

**Affiliations:** 1 School of Economics, University of the Philippines, Diliman, Quezon City, Philippines; 2 Department of Applied Economics, Erasmus School of Economics, Erasmus University Rotterdam, Rotterdam, Netherlands; 3 Tinbergen Institute, Amsterdam, Netherlands; 4 University of Lausanne, Lausanne, Switzerland; Universidade Federal do Rio Grande do Sul, BRAZIL

## Abstract

**Objective:**

Estimate associations between the health-related quality of life (HRQoL) and adiposity in a low-income population.

**Methods:**

In a cluster random sample of 3796 Filipinos aged 40–70 years in Nueva Ecija province, we measured body mass index (BMI), waist circumference (WC), waist-to-hip ratio (WHR), and six dimensions of HRQoL using the 20-item Short Form Health Survey. We stratified by sex and used nonparametric regression to graph mean HRQoL in each dimension by BMI, WC, and WHR. We used ordinary least squares regression to estimate differences in each HRQoL dimension by categories of BMI, WC, and WHR adjusted for sociodemographic characteristics and smoking.

**Results:**

Mean HRQoL was lowest for health perception (Males: 67.5 (SD = 15.9); Females: 66.7 (15.8)) and highest for role functioning (Males: 97.5 (12.9); Females: 97.4 (13.3)). Mean (SD) values of BMI, WC, and WHR were 22.1 (3.6), 84.8 cm (9.5), and 0.9 (0.1), respectively for males, and 23.7 (4.2), 86.5 cm (10.2), and 0.9 (0.1), respectively, for females. There was no evidence that higher BMI was associated with lower HRQoL. Adjusted mean social functioning was 4.92 (p = 0.076) higher for males with high BMI risk (8.6% prevalence) compared with acceptable BMI risk (50.3%). Mean social functioning was 3.61 (p = 0.012) and 5.48 (p = 0.017) lower for females with high WC (44.7%) and WHR (83.1%), respectively, compared with those with low WC (23.8%) and WHR (3.6%). Mean physical functioning was lower by 2.70 (p = 0.204) and 1.07 (p = 0.198) for males and females, respectively, with high compared with low WC. Mean physical functioning was 3.93 (p = 0.037) lower for males with high (7.6%) compared with low (38.8%) WHR. Mean role functioning was 1.09 (p = 0.124) and 2.46 (p = 0.158) lower for males with borderline and high WHR, respectively.

**Conclusions:**

There is discordance between future adiposity-related health risk and current experience of HRQoL.

## Introduction

Obesity is an established risk factor for multiple diseases [1–4]. The lag between exposure to obesity and the onset of disease should make prevention possible. But prevention will be difficult if overweight people who are at risk of disease are unaware of any developing health problem. Awareness of health deterioration only after becoming obese, and not at lower thresholds, may come too late to change behavior or avert the risk.

Health-related quality of life [HRQoL] measures an individual’s perception of their health in multiple dimension [5, 6]. It allows examination of whether people whose weight puts them at increased risk of disease perceive themselves to be in worse health. The evidence is mixed [7–16]. Associations between HRQoL and adiposity have been found to be nonlinear [7, 12, 16, 17], differ across dimensions of HRQoL [9, 14, 15], and vary with the measure of adiposity [14]. Associations also differ across populations defined by country [12, 15, 18], ethnicity [8], and sex [9, 12, 14, 16, 18–20].

Most of the evidence on the association, if any, between HRQoL and adiposity comes from high-income countries in North America and Europe [7, 9, 10, 21]. Evidence from East Asia also tends to be for high-income countries [12, 19–22]. Lack of evidence on the HRQoL-adiposity association in low- and middle-countries (LMIC) in Asia, and elsewhere, is limiting given the rising prevalence of obesity in these countries [23, 24]. Without this evidence, it is more difficult to design and target disease prevention programs that are critical to protecting resource-constrained health systems with underdeveloped chronic disease management in primary care. Evidence on the existence, nature, and strength of any HRQoL-adiposity association in Asian LMIC is potentially particularly valuable because disease risks may occur at lower adiposity thresholds in Asian populations [25, 26].

Most studies of the HRQoL-adiposity relationship have used the RAND 36-item Short Form Health Survey (SF-36) to measure HRQoL and the body mass index (BMI) to measure adiposity [7, 11, 12, 15, 17–19, 27–29]. Many found a negative relationship between physical health, measured by the SF-36 physical component score, and BMI [11, 12, 15, 17, 18–21]. Evidence is more mixed on the relationship between the mental health component score and BMI [7, 12, 14, 15, 17, 18, 20, 21]. Some studies found that overweight and moderately obese people reported better mental health [7, 14, 15, 17]. There is some evidence from Asia of this *obesity paradox*: class 1 obesity has been found to be associated with better mental health, physical functioning, role functioning, social functioning, mental health, vitality, and general health [15, 17], and mild abdominal obesity was found to be associated with better physical functioning and vitality among women [29]. People who are underweight have often been found to have the lowest HRQoL [9, 15], resulting in an inverse U-shaped relationship between some dimensions of HRQoL and BMI [7, 17].

The focus on BMI, with some exceptions [20], is limiting because central adiposity is a more relevant risk factor for some diseases [30–32]. Since measures of adiposity are not perfectly correlated [33–38] they do not necessarily all have the same association with HRQoL. For example, if HRQoL were to decrease as WHR increased but it was relatively unresponsive to BMI, perhaps because of greater self-awareness of high WHR, then it may be easier to convince people to adopt healthier habits when they are close to a WHR risk threshold than when they are close to a BMI risk threshold.

The aim of this study was to test for and estimate the magnitude of any association between HRQoL and adiposityin a low-income population. It aimed to explore heterogeneity in the association by sex, dimension of HRQoL, and the measure of adiposity. Further, we aimed to examine nonlinearity in the relationship across the distribution of each adiposity measure.

## Methods

### Participants

In January-May 2018, we conducted a survey in Nueva Ecija, a mainly rural province about 180 km north of Metro Manila in the Philippines. Compared with the whole country, the province had only a slightly higher poverty rate (22.6% vs 21.6%) [39], a lower human development index (0.603 vs 0.711) [40], similar income inequality (Gini 0.4278 vs 0.4266) [39], and slightly higher prevalence of overweight/obesity (32.7% vs 31.3%) [41]. The survey was the baseline of a randomized experiment designed to evaluate primary prevention of cardiovascular diseases (CVD) [42]. This paper reports an observational study of the association between HRQoL and adiposity at baseline prior to assignment to any intervention. This study was not the main purpose of the randomized experiment.

After stratifying by urban/rural location, we randomly sampled 304 barangays from 849 in the province. A barangay is the smallest administrative unit in the country. Within each sampled barangay, we selected 12 households by random interval sampling. Within each household, we randomly selected one person aged 40–70 years old.

To serve the main purpose of the randomized experiment, individuals who were not in the target population for primary prevention of CVD were screened out of the sample. Excluded were those who (a) had a diagnosis of diabetes or heart disease, (b) had suffered a heart attack or stroke, or (c) were taking medication for hypertension. Also excluded were those with a health condition that prevented measurement of height, weight, or blood pressure. Each excluded participant was replaced with another randomly selected person aged 40–70 years from the same household, or, if needed, from another randomly selected household in the same barangay. We used data from the baseline survey, which was conducted in one session by trained enumerators in participants’ homes, only and so did not lose any participants through attrition. The sample was representative of the target population for primary CVD prevention in Nueva Ejica,

The study protocol of the randomized experiment that generated the data used here obtained ethical approval from the Institutional Review Boards of the UPecon Foundation (Philippines) in June 2017 and Canton de Vaud (Switzerland) in July 2017. Written informed consent was obtained from all study participants. We did not prespecify the statistical analysis for this study.

### Health-related quality of life

The study objective was to determine whether HRQoL varied with exposure to adiposity. We measured HRQoL using self-reports to the 20-Item Short Form Health Survey (SF-20) that consists of 20 questions in six dimensions: physical functioning (6 items), role functioning (2 items), social functioning (1 item), mental health (5 items), current health perceptions (5 items), and pain (1 item) (S1 Table in S1 File) [43]. The items were scored using a validated algorithm [43]. Scores were transformed to the 0–100 scale, with 0 indicating the worst and 100 the best HRQoL for each of the six dimensions.

### Adiposity

After participants completed the SF20 instrument, trained enumerators measured the weight and height of each participant. Weight in kilograms (kg) was measured using a digital scale (Tanita HD-32 brand). If a participant could not stand on the scale, the interview was terminated. Bulky clothing, footwear, accessories, and artificial limbs were removed before standing on the scale. If an artificial limb could not be removed, the participant was asked to report its approximate weight, which was subtracted from the participant’s measured weight. Height in meters (m) was measured using a tape measure with a participant standing, without footwear, straight against a wall with heals, buttocks, and back against the wall.

We calculated BMI (= weight (kg) / height (m)^2^) and categorized participants using WHO thresholds for Asian populations: <18.5 = underweight, 18.5–22.9 = acceptable risk, 23–27.4 = increased risk, and ≥ 27.5 = high risk [44]. In sensitivity analyses, we used the international standard categories: <18.5 = underweight, 18.5–24.9 = normal weight, 25–29.9 = overweight, and ≥ 30.0 = obese.

Enumerators measured waist circumference (WC) and WHR following standard procedures. Each participant was asked to remove bulky clothing before wrapping a tape measure around their waist near the navel. They were asked to breath normally and make a normal expiration, after which WC was measured. Then, the participant was instructed to wrap the tape around the maximum circumference of the buttocks. After ensuring the tape was snug and horizontal, hip circumference was measured.

We used WHO thresholds for Asian populations to define low, borderline, and high WC and WHR [45, 46]. For WC, the respective thresholds are <80 cm, 80–87.9 cm, and ≥88 cm for females, and <90 cm, 90–101.9 cm, and ≥102 cm for males. For WHR, the respective thresholds are <0.8 cm, 0.8–0.84 cm, and ≥0.85 cm for females, and <0.9 cm, 0.9–0.99 cm, and ≥1.0 cm for males.

### Covariates

Multivariable analyses of associations between dimensions of HRQoL and adiposity measures controlled for sociodemographic characteristics. At the participant level, these were: age (5-year intervals 40–44, etc.), marital status, whether graduated from high school, whether employed, and whether smokes. At the household level, control was for urban/rural location and wealth quintile group. Wealth was proxied by the first principal component of a factor analysis of house building materials, water source, sanitation, possessions of durable assets, and participation in the government conditional cash transfer program [47].

In sensitivity analyses, we additionally controlled for the participant having reported a diagnosed chronic condition (cancer, lung disease, bone diseases, neurological or psychiatric problems) and a first-degree relative having had a diagnosed chronic condition (hypertension, diabetes, high cholesterol, heart disease, stroke, or malignant tumor). All covariates were reported by the participant.

### Statistical analyses

Interest was in whether overweight people, who objectively faced higher disease risks, reported experiencing worse health. In cross-sectional, descriptive analyses, we regressed HRQoL on measures of adiposity to estimate associations between these variables.

We stratified all analyses by sex. We used nonparametric local linear bivariate regression [48] to estimate and graph the conditional mean of each dimension of HRQoL as a function of each continuous measure of adiposity. We used the Epanechnikov kernel function and selected the bandwidth using the plugin estimator of the asymptotically optimal constant bandwidth [48].

We used ANOVA (F-test) to test that the mean of each dimension of HRQoL was equal across categories of each adiposity measure. We used ordinary least squares regression to estimate differences in the mean of each HRQoL dimension across adiposity categories conditional on covariates. For each sex and each dimension of HRQoL, we estimated three regressions. Each of these regressions included categories of BMI, WC, or WHR. The main results were estimated with control for all sociodemographics and smoking. In sensitivity analyses, we estimated two alternative models. One controlled only for age. The other controlled for the participant’s and their family’s history of chronic conditions, in addition to all the covariates used to produce the main results.

We adjusted 95% confidence intervals (CI) of the regression estimates for the clustered sample design. All analyses were conducted using Stata ® version 17.

## Results

Of 4126 randomly selected individuals, 37 were ineligible, 137 refused, and 156 did not complete the interview. The analysis sample of 3796 individuals consisted of 1298 (34.2%) males and 2498 (65.8%) females.

Table 1 shows the characteristics of this sample by sex. Mean ages were 52.6 and 51.2 years for males and female, respectively. Most participants (> 55%) had not graduated from high school and almost three quarters were located in rural areas. About 85% of males, but only 45% of females, were employed. The percentage of smokers was 58% for males and 12% for females. Only 21% of males and 23% of females reported being diagnosed with a chronic illness, but over 70% had a family member with such an illness.

**Table 1:** Participant characteristics by sex.

	Male				Female			
	(N = 1,298)				(N = 2,498)			
	*mean*	*(sd)*	*n*	*(%)*	*mean*	*(sd)*	*n*	*(%)*
*Adiposity measures*								
Body mass index (kg/m^2^)	22.1	(3.6)			23.7	(4.2)		
Underweight			176	(13.6)			227	(9.1)
Acceptable risk			653	(50.3)			922	(36.9)
Increased risk			358	(27.6)			918	(36.7)
High risk			111	(8.6)			431	(17.3)
Waist circumference (cm)	84.8	(9.5)			86.5	(10.2)		
Low			925	(71.3)			595	(23.8)
Borderline			306	(23.6)			787	(31.5)
High			67	(5.2)			1,116	(44.7)
Waist-to-hip ratio	0.9	(0.1)			0.9	(0.1)		
Low			503	(38.8)			91	(3.6)
Borderline			696	(53.6)			332	(13.3)
High			99	(7.6)			2,075	(83.1)
*Health-related quality of life (SF-20)*								
Physical	93.5	(14.7)			92.8	(15.3)		
Role	97.5	(12.9)			97.4	(13.3)		
Social	82.8	(28.4)			82.5	(28.3)		
Mental	86.1	(12.0)			84.7	(12.3)		
Health Perception	67.5	(15.9)			66.7	(15.8)		
Pain	85.7	(20.8)			84.3	(21.6)		
* Covariates*								
Age	52.6	(8.6)			51.2	(8.2)		
Married								
No			227	(17.5)			550	(22.0)
Yes			1,071	(82.5)			1,948	(78.0)
High school graduate								
No			764	(58.9)			1,386	(55.5)
Yes			534	(41.1)			1,112	(44.5)
Employed								
No			193	(14.9)			1,384	(55.4)
Yes			1,105	(85.1)			1,114	(44.6)
Urban								
No			996	(76.7)			1,797	(71.9)
Yes			302	(23.3)			701	(28.1)
Wealth quintile group								
Poorest			276	(21.3)			484	(19.4)
2^nd^ poorest			281	(21.6)			478	(19.1)
Middle			256	(19.7)			503	(20.1)
2^nd^ richest			238	(18.3)			521	(20.9)
Richest			247	(19.0)			512	(20.5)
Smokes currently								
No			550	(42.4)			2,191	(87.7)
Yes			748	(57.6)			307	(12.3)
History of chronic illness ^a^								
No			1,025	(79.0)			1,914	(76.6)
Yes			273	(21.0)			584	(23.4)
Family history of chronic illness ^b^								
No			386	(29.7)			664	(26.6)
Yes			912	(70.3)			1,834	(73.4)

^a^ Cancer or malignant tumor, chronic lung disease (such as asthma, chronic obstructive pulmonary disease/chronic bronchitis or other chronic lung problems), arthritis or rheumatism, osteoporosis or other bone diseases, or any neurological, or psychiatric problems, such as depression, unipolar/bipolar disorders, convulsions etc.

^b^ Hypertension, high blood sugar/diabetes, high cholesterol, heart attack, heart disease, stroke, cancer or malignant tumor

In the sample, mean BMI was 22.1 for males and 23.7 for females. On the basis of BMI, 27.5% of males and 36.7% of females were at increased risk, while 8.6% and 17.3%, respectively, were at high risk. Sample mean WC was 84.8 cm for males and 86.5 cm for females. Only 5.2% of males, but 44.7% of females, were at high risk on the basis of WC. Mean WHR was 0.9 for both sexes. Only 7.6% of males, but 83.1% of females, were classified as high risk for WHR.

Sample mean HRQoL was highest in the role functioning dimension (Males: 97.5; Females: 97.4) and lowest in the health perception dimension (Males: 67.5; Females: 66.7) for both sexes. In most dimensions, mean HRQoL was slightly lower for female participants.

**Fig 1:**
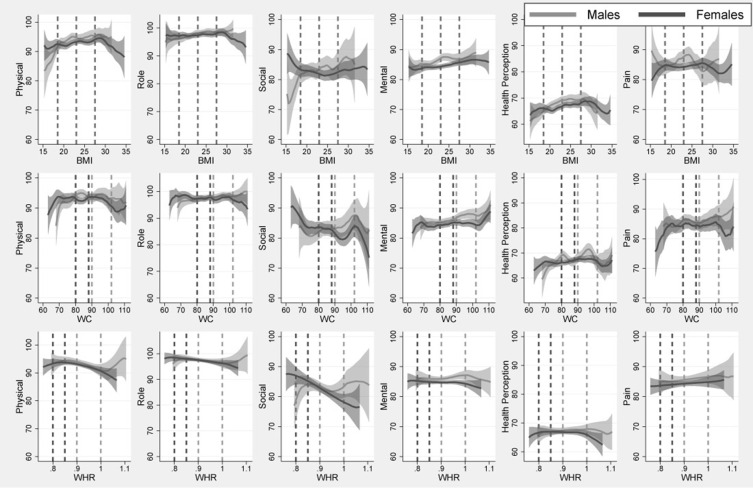
Means of health-related quality of life dimensions by body mass index (BMI), waist circumference (WC), waist-to-hip ratio (WHR), and by sex. *Notes*. Each graph shows a conditional mean function estimated by local linear bivariate regression using the Epanechnikov kernel function and the plugin estimator of the asymptotically optimal constant bandwidth. Shading shows 95% confidence intervals. Dashed vertical lines indicate increased and high risk for BMI, and borderline and high for WC and WHR.

Fig 1 shows, for each sex, the estimated conditional mean of each dimension of HRQoL as a function of BMI (top), WC (middle), and WHR (bottom). Shading indicates 95% CI. From the left, the first column shows physical functioning, which was higher at higher BMI through the thresholds that define increased risk (BMI>22.9) and high risk (BMI>27.4). Above the obesity threshold (BMI>29.9), physical functioning was lower for individuals with higher BMI. Physical functioning was lower for women with higher WC only once WC was above the high risk threshold. For males, physical function was lower for individuals with higher WC even before the high risk threshold, although the 95% CI is wide in this region. For both sexes, physical functioning was lower for individuals with higher WHR, including through the thresholds for borderline and high risk. There was an upturn in physical functioning at very high WHR for males, but the data are very sparse in this region.

For role functioning the patterns are similar. This dimension of HRQoL was also not lower, and for males it was higher, at higher values of both BMI and WC through the thresholds that define increased/borderline risk and high risk. Again, the relationship with WHR was negative.

For social functioning, the relationships differ by sex. For the most part and through the relevant thresholds, males with higher BMI and WC had better social functioning. This dimension of HRQoL was lower for females with higher values of WC and WHR.

Mental health, particularly of males, was better at higher BMI and WC. The (positive) health perception of males peaked before BMI and WC reached the respective thresholds that define high risk. Only above these thresholds did females with higher BMI and WC have perceptions of worse health. There was no clear relationship between pain and BMI. Particularly for males, HRQoL in the domain of pain was higher (less pain) for individuals with higher WC and WHR.

Table 2 gives the mean of each dimension of HRQoL by categories of BMI, WC, and WHR, and by sex. Males in categories of higher BMI had significantly better physical functioning (P < 0.0001). But males in categories of higher WHR had significantly worse physical functioning (P < 0.05). Male role functioning, social functioning, and mental health were all significantly better in categories of higher BMI (P < 0.05, P < 0.1, and P < 0.05, respectively). Mental health of males was also better in categories of higher WC (P < 0.05). The health perception of males peaked at the increased risk category of BMI and the borderline category of WC.

**Table 2:** Means of health-related quality of life dimensions by categories of body mass index (BMI), waist circumference (WC), and waist-to-hip ratio (WHR), and sex.

	Physical				Role				Social				Mental				Health Perception				Pain			
	Mean	95% CI		P	Mean	95% CI		P	Mean	95% CI		P	Mean	95% CI		P	Mean	95% CI		P	Mean	95% CI		P
**Males**																								
Body mass index				0.0000				0.0269				0.0796				0.0465				0.0000				0.4695
Underweight	87.1	[84.1,	90.1]		95.3	[92.5,	98.1]		79.3	[74.9,	83.7]		84.8	[83.0,	86.6]		62.6	[60.2,	65.1]		84.9	[81.8,	88.0]	
Acceptable risk	94.2	[93.1,	95.3]		97.2	[96.2,	98.3]		82.4	[80.2,	84.5]		85.6	[84.7,	86.6]		67.6	[66.4,	68.8]		85.6	[84.0,	87.2]	
Increased risk	94.7	[93.4,	96.1]		98.7	[97.8,	99.5]		83.7	[80.7,	86.7]		87.2	[86.1,	88.4]		69.5	[68.0,	71.1]		87.0	[84.9,	89.2]	
High risk	95.4	[93.3,	97.5]		98.6	[97.1,	100.2]		87.9	[83.4,	92.4]		87.6	[85.4,	89.8]		68.0	[65.1,	70.9]		84.0	[79.6,	88.3]	
Waist circumference				0.5898				0.1210				0.2968				0.0231				0.0463				0.7562
Low	93.4	[92.4,	94.3]		97.0	[96.1,	97.9]		82.1	[80.2,	83.9]		85.6	[84.8,	86.4]		66.9	[65.9,	68.0]		85.6	[84.2,	86.9]	
Borderline	94.1	[92.5,	95.6]		98.8	[97.9,	99.6]		84.2	[81.1,	87.3]		87.2	[85.9,	88.5]		69.4	[67.7,	71.1]		85.9	[83.6,	88.3]	
High	92.2	[88.1,	96.2]		97.8	[94.4,	101.1]		86.3	[80.4,	92.2]		88.8	[86.1,	91.4]		66.4	[62.7,	70.1]		87.5	[82.5,	92.4]	
Waist-to-hip ratio				0.0460				0.2137				0.2728				0.4687				0.7815				0.8643
Low	94.5	[93.4,	95.7]		98.2	[97.2,	99.1]		84.1	[81.8,	86.4]		85.6	[84.6,	86.7]		67.6	[66.2,	68.9]		85.4	[83.5,	87.2]	
Borderline	93.1	[92.0,	94.2]		97.2	[96.2,	98.2]		81.6	[79.4,	83.8]		86.5	[85.6,	87.4]		67.6	[66.4,	68.8]		85.9	[84.4,	87.5]	
High	90.8	[87.0,	94.6]		96.0	[92.3,	99.6]		84.4	[79.1,	89.8]		86.2	[84.1,	88.4]		66.4	[63.3,	69.5]		86.3	[82.3,	90.2]	
**Females**																								
Body mass index				0.6225				0.9367				0.4236				0.0182				0.1405				0.7874
Underweight	91.8	[89.6,	94.0]		97.2	[95.4,	99.1]		84.4	[81.1,	87.7]		83.0	[81.4,	84.6]		65.2	[63.1,	67.4]		83.1	[80.1,	86.1]	
Acceptable risk	92.6	[91.6,	93.6]		97.3	[96.4,	98.2]		82.4	[80.6,	84.2]		84.4	[83.6,	85.2]		66.1	[65.1,	67.1]		84.3	[82.9,	85.7]	
Increased risk	93.1	[92.2,	94.1]		97.6	[96.8,	98.5]		81.6	[79.7,	83.5]		84.8	[84.0,	85.6]		67.3	[66.3,	68.3]		84.7	[83.3,	86.1]	
High risk	93.1	[91.5,	94.6]		97.3	[96.2,	98.5]		83.8	[81.2,	86.4]		86.0	[84.9,	87.2]		67.5	[66.0,	68.9]		84.2	[82.1,	86.3]	
Waist circumference				0.7992				0.7808				0.0410				0.1569				0.2386				0.7993
Low	93.1	[91.9,	94.3]		97.7	[96.7,	98.7]		84.5	[82.4,	86.5]		84.0	[82.9,	85.0]		66.0	[64.8,	67.3]		83.8	[82.0,	85.6]	
Borderline	92.5	[91.5,	93.6]		97.5	[96.5,	98.4]		83.2	[81.2,	85.1]		84.6	[83.8,	85.5]		66.4	[65.2,	67.5]		84.5	[83.0,	86.0]	
High	92.8	[92.0,	93.7]		97.2	[96.4,	98.0]		81.0	[79.3,	82.8]		85.2	[84.4,	85.9]		67.3	[66.3,	68.2]		84.5	[83.2,	85.7]	
Waist-to-hip ratio				0.1305				0.2788				0.0007				0.2425				0.4328				0.8904
Low	91.8	[88.3,	95.2]		97.3	[94.1,	100.4]		87.5	[83.0,	92.0]		84.5	[82.0,	86.9]		65.7	[62.6,	68.8]		83.5	[78.6,	88.4]	
Borderline	94.3	[93.0,	95.7]		98.5	[97.4,	99.6]		87.2	[84.6,	89.8]		85.8	[84.4,	87.1]		67.6	[66.0,	69.3]		84.7	[82.3,	87.1]	
High	92.6	[91.9,	93.3]		97.2	[96.7,	97.8]		81.6	[80.3,	82.8]		84.5	[84.0,	85.1]		66.6	[65.9,	67.3]		84.3	[83.4,	85.2]	

*Notes*. P is the p value from an F test (ANOVA) of equal means across the respective adiposity categories.

For females, there were fewer significant differences. Females in higher categories of WC and WHR had significantly worse social functioning (P < 0.05 and P < 0.001, respectively). Female mental health was significantly better in categories of higher BMI (P < 0.05). These results are robust to replacing the Asian population BMI categories with the international standard categories, with the exception that obese males and females have lower physical functioning (S2 Table in S1 File).

**Fig 2:**
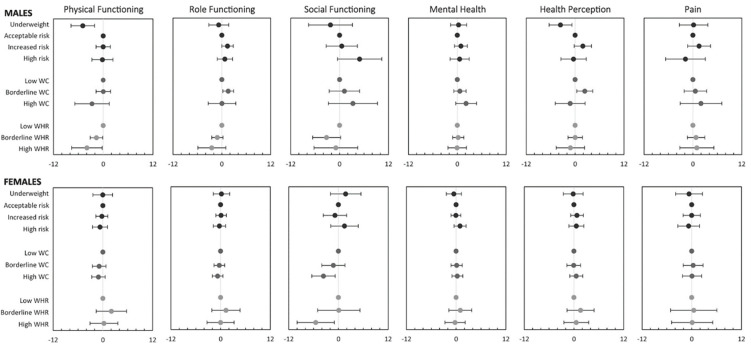
Adjusted differences in means of health-related quality of life dimensions between categories of body mass index (BMI), waist circumference (WC), and waist-to-hip ratio (WHR) by sex. *Notes*. Multivariable ordinary least squares regression estimates. For each sex and HRQoL dimension, three regressions were estimated. One included BMI categories, another included WC categories, and the third included WHR categories. References categories are indicated by a dot at 0 with no whiskers that elsewhere indicate 95% CI. All regressions included the covariates in Table 1, with age entered as indicators of 5-year intervals and participant and family history of chronic condition excluded. Estimates in table format in S3 Table in S1 File.

Fig 2 shows multivariable regression estimates of differences in the conditional mean of each dimension of HRQoL across the categories of BMI, WC, and WHR (S3 Table in S1 File for estimates in table format). After controlling for covariates, underweight males continued to have significantly lower physical functioning (-4.93, P < 0.01), while men at increased and high risk according to their BMI did not have significantly different physical functioning from those with BMI in the acceptable risk interval. The physical functioning of men with borderline (-1.63) and high (-3.93) WHR was significantly worse than the functioning of those with low WHR (P < 0.05). Role functioning was significantly higher for males at increased risk by BMI (1.40, P < 0.1) and borderline WC (1.58, P < 0.05) compared with those at acceptable BMI risk and low WC, respectively. After adding controls, role functioning continued to be worse for males with borderline and high WHR, although the differences were not significant. Male social functioning and mental health remained higher in categories of higher BMI and WC, while health perceptions still peaked at increased risk by BMI and borderline WC, although not all of these differences were significant (P < 0.1) after adding controls.

After controls were added, female social functioning remained significantly worse in categories of higher WC (-3.61, P<0.05) and WHR (-5.48, P < 0.05). The multivariable regression results were robust to adding participant and family history of chronic conditions to the controls and to estimating a restricted model that only controlled for age (S1 Fig and S4 and S5 Tables in S1 File).

## Discussion

In a predominantly low-income, rural province of the Philippines, we did not find that people who were at disease risk according to measures of adiposity had lower health-related quality of life, providing further evidence on the nuanced association between HRQoL and adiposity in Asian population [25, 26]. There was some variation in the association between HRQoL and adiposity by sex, adiposity measure, and dimension of HRQoL. For both sexes, we found no evidence that people with a BMI that put them at increased or high risk had lower HRQoL in any dimension. In fact, particularly for males, there was some evidence that people at high BMI risk had better social functioning, consistent with findings in the USA and East Asia [7, 14, 15, 17]. Similar to findings in the USA and China, underweight males had lower physical functioning and health perception [9, 15].

Risks defined by WC and WHR were associated with lower HRQoL more often than was observed for BMI risks. Physical functioning was lower for people at high risk by WC and for males at high risk by WHR. Males at borderline risk and high risk by WHR also had lower role functioning. Females at high risk by WC and WHR had lower social functioning. At least in the sample, males at high risk by WC and WHR had lower health perceptions. Otherwise, there was no evidence of higher adiposity risk being associated with lower HRQoL. In fact, males at high risk by BMI had better social functioning, and males at high risk by WC had better social functioning and mental health,which are similar to previous findings [7, 14, 15, 17].

These findings reveal that Filipinos whose body weight places them at higher disease risk are generally unaware of any health deterioration across a number of dimensions of related quality of life. Consequently, it may be difficult to persuade these people that their health is truly at risk. If they are continuing to function as well as, or even better than, people who are not overweight, then they must accept an abstract argument that they are exposed to higher risk of succumbing to disease in the future without direct experience of health problems giving them reason to believe that the risk will indeed materialize.

It may be important to take this discordance between risk to future health and experience of current health into account in the design of effective prevention programs. For example, information on disease risks associated with overweight could be accompanied by a message that currently feeling in good health does not guarantee the absence of weight-related health problems in the future. Such messages may need to be stronger for less educated individuals who may have less capacity to interpret risks and a greater tendency to behave in response to experiences rather than information.

We found that risks defined by WC and WHR were more often associated with lower HRQoL than were BMI risks. This is consistent with some evidence that high WC and WHR represent greater disease risks than high BMI [30–32]. If the experience of health problems is more closely associated with the types of adiposity that give exposure to the highest objective risks, then there is greater scope to convince people to take steps to reduce their waist size.

We should not, however, exaggerate the heterogeneity by type of adiposity. High WC and WHR was not strongly associated with lower HRQoL in most dimensions. Even people with large waists will need to be convinced that the fact that they are not experiencing worse health than slimmer people does not mean that they can be relaxed with respect to their future health prospects. Further men with high WC experienced better social functioning and mental health, and so it may be particularly difficult to convince males of the risks associated with central obesity. Their body size and shape could well reflect their enjoyment of life’s pleasures. With little or no experience of offsetting health problems, there will be less motivation to abstain from excessive eating and drinking. On the other hand, women with high WHR had worse social functioning, which could potentially motivate them to lose weight. Finally, while disease risks associated with high BMI may be lower than those for high WC and high WHR, high BMI is certainly not without any risk to health. Prevention programs need to work at changing any perceptions that only abdominal obesity is a risk to health.

The main limitation of the study is that it collected data from only one province of the Philippines. The sample was representative of that province. But we cannot claim that the results are representative of the whole country. However, the study setting was reasonably typical of the socioeconomic conditions and rural setting in which much of the lower-income Filipino population lives. Studies of such populations that measure both adiposity and HRQoL are relatively rare.

The evidence we provided of a general lack of association between HRQoL and measures of adiposity is relatively rare in a low-income setting. It can help in motivating and designing health promotion programs that aim to prevent the disease burden that threatens to accumulate from the rising prevalence of obesity in LMIC. Our central finding deserves attention precisely because it is negative. Overweight people at high risk of future disease who are not currently experiencing health problems have less reason to change their behavior. They will need greater external encouragement to do so.

## Supporting information

S1 File (DOCX)
