# Supplementary material for: Associations between health-related quality of life and measures of adiposity among Filipino adults
Source: PLoS One. 2022 Oct 26;17(10):e0275798. doi: 10.1371/journal.pone.0275798 (PMC9605333; doi:10.1371/journal.pone.0275798)
Supplement: S1 File — (DOCX) [file pone.0275798.s001.docx]

**Associations between health-related quality of life and measures of adiposity among Filipino adults aged 40-70 years**

Joseph Capuno^1,*^, Aleli Kraft^1^, Kayleen Gene Calicdan^1^, Owen O’Donnell^2^

^1^School of Economics, University of the Philippines, Diliman, Quezon City 1101, Philippines

^2^ Department of Applied Economics, Erasmus School of Economics, Erasmus University Rotterdam, P.O. Box 1738, 3000, DR Rotterdam, Netherlands; Tinbergen Institute; University of Lausanne

***Corresponding author**: jjcapuno@up.edu.ph (JC)

**Supplementary Material**

**Associations between health-related quality of life and measures of adiposity among Filipino adults aged 40-70 years**

**Table of contents**

| Table S1. SF-20 questions by HRQoL dimension |
| --- |
| Table S2. Means of health-related quality of life dimensions by standard international categories of body mass index (BMI) and sex |
| Table S3. Adjusted differences in means of six HRQoL dimensions by BMI, Waist Circumference and Waist-Hip Ratio categories, and by sex |
| Table S4. Adjusted differences in means of six HRQoL dimensions by BMI, Waist Circumference and Waist-Hip Ratio categories, alternative specifications, Males |
| Table S5. Adjusted differences in means of six HRQoL dimensions by BMI, Waist Circumference and Waist-Hip Ratio categories, alternative specifications, Females |
| Figure S1. Adjusted differences in means of health-related quality of life dimensions between categories of body mass index (BMI), waist circumference (WC), and waist-to-hip ratio (WHR) by sex |

| **Table S1. SF-20 questions by HRQoL dimension** | | | |
| --- | --- | --- | --- |
| HRQoL Dimension | Question | Response categories | Item No. |
| Physical Functioning | For how long (if at all) has your health limited you in each of the following activities?   - The kinds or amounts of vigorous activities you can do, like lifting heavy objects, running or participating strenuous sports | \| 1 \| Limited for more than 3 months \| \| --- \| --- \| \| 2 \| Limited for 3 months or less \| \| 3 \| Not limited at all \| | 2 |
|  | - The kinds or amounts of moderate activities you can do, like moving a table, carrying groceries, or bowling |  | 3 |
|  | - Walking uphill or climbing a few flights of stairs |  | 4 |
|  | - Bending, lifting, or stooping |  | 5 |
|  | - Walking one block |  | 6 |
|  | - Eating, dressing, bathing, or using the toilet |  | 7 |
| Role Functioning | Does your health keep you from working at a job, doing work around the house, or going to school? | \| 1 \| YES, for more than 3 months \| \| --- \| --- \| \| 2 \| YES, for 3 months or less \| \| 3 \| NO \| | 9 |
|  | Have you been unable to do certain kinds or amounts of work, housework, or schoolwork because of your health? |  | 10 |
|  | For the following questions, choose one answer that comes closest to the way you have been feeling during the past month. |  |  |
| Social Functioning | How much of the time, during the past month, has your health limited your social activities (like visiting with friends or close relatives)? | \| 1 \| All of the time \| \| --- \| --- \| \| 2 \| Most of the time \| \| 3 \| A good bit of the time \| \| 4 \| Some of the time \| \| 5 \| A little of the time \| \| 6 \| None of the time \| | 11 |
| Mental Health | How much of the time, during the past month, have you been a very nervous person? |  | 12 |
|  | During the past month, how much of the time have you felt calm and peaceful? |  | 13 |
|  | How much of the time, during the past month, have you felt downhearted and blue? |  | 14 |
|  | During the past month, how much of the time have you been a happy person? |  | 15 |
|  | How often, during the past month, have you felt so down in the dumps that nothing could cheer you up? |  | 16 |
| Health Perception | In general, would you say your health is: | \| 1 \| Excellent \| \| --- \| --- \| \| 2 \| Very good \| \| 3 \| Good \| \| 4 \| Fair \| \| 5 \| Poor \| | 1 |
|  | Please answer whether each of the following statements is true or false for you.   - “I am somewhat ill” | \| 1 \| Definitely true \| \| --- \| --- \| \| 2 \| Mostly true \| \| 3 \| Not sure \| \| 4 \| Mostly false \| \| 5 \| Definitely false \| | 17 |
|  | - “I am as healthy as anybody I know” |  | 18 |
|  | - “My health is excellent” |  | 19 |
|  | - “I have been feeling bad lately” |  | 20 |
| Pain | How much bodily pain have you had during the past 4 weeks? | \| 1 \| None \| \| --- \| --- \| \| 2 \| Very mild \| \| 3 \| Mild \| \| 4 \| Moderate \| \| 5 \| Severe \| \| 6 \| Very severe \| | 8 |

| **Table S2. Means of health-related quality of life dimensions by standard international categories of body mass index (BMI) and sex** | | | | | | | | | | | | | | | | | | | | | | | | | | | | | | |
| --- | --- | --- | --- | --- | --- | --- | --- | --- | --- | --- | --- | --- | --- | --- | --- | --- | --- | --- | --- | --- | --- | --- | --- | --- | --- | --- | --- | --- | --- | --- |
|  |  | **Physical** | | | |  | **Role** | | | |  | **Social** | | | |  | **Mental** | | | |  | **Health Perception** | | | |  | **Pain** | | | |
|  |  | Mean | 95% CI | | P |  | Mean | 95% CI | | P |  | Mean | 95% CI | | P |  | Mean | 95% CI | | P |  | Mean | 95% CI | | P |  | Mean | 95% CI | | P |
| **Males** |  |  |  |  |  |  |  |  |  |  |  |  |  |  |  |  |  |  |  |  |  |  |  |  |  |  |  |  |  |  |
| Body Mass Index |  |  |  |  | 0.0000 |  |  |  |  | 0.0700 |  |  |  |  | 0.2566 |  |  |  |  | 0.2747 |  |  |  |  | 0.0000 |  |  |  |  | 0.6054 |
| Underweight |  | 87.1 | [84.1, | 90.1] |  |  | 95.3 | [92.5, | 98.1] |  |  | 79.3 | [74.9, | 83.7] |  |  | 84.8 | [83.0, | 86.6] |  |  | 62.6 | [60.2, | 65.1] |  |  | 84.9 | [81.8, | 88.0] |  |
| Normal |  | 94.2 | [93.3, | 95.2] |  |  | 97.6 | [96.7, | 98.4] |  |  | 82.9 | [81.0, | 84.8] |  |  | 86.2 | [85.4, | 87.0] |  |  | 68.1 | [67.0, | 69.1] |  |  | 86.3 | [84.9, | 87.7] |  |
| Overweight |  | 95.4 | [93.9, | 97.0] |  |  | 98.6 | [97.5, | 99.8] |  |  | 84.3 | [80.8, | 87.8] |  |  | 86.6 | [85.0, | 88.2] |  |  | 69.6 | [67.6, | 71.5] |  |  | 84.4 | [81.7, | 87.1] |  |
| Obese |  | 93.8 | [89.3, | 98.3] |  |  | 98.6 | [95.7, | 101.5] |  |  | 86.9 | [78.2, | 95.5] |  |  | 88.5 | [85.2, | 91.7] |  |  | 63.1 | [58.5, | 67.7] |  |  | 86.3 | [78.3, | 94.2] |  |
|  |  |  |  |  |  |  |  |  |  |  |  |  |  |  |  |  |  |  |  |  |  |  |  |  |  |  |  |  |  |  |
| **Females** |  |  |  |  |  |  |  |  |  |  |  |  |  |  |  |  |  |  |  |  |  |  |  |  |  |  |  |  |  |  |
| Body mass index |  |  |  |  | 0.1494 |  |  |  |  | 0.3200 |  |  |  |  | 0.7469 |  |  |  |  | 0.0034 |  |  |  |  | 0.1085 |  |  |  |  | 0.2399 |
| Underweight |  | 91.8 | [89.6, | 94.0] |  |  | 97.2 | [95.4, | 99.1] |  |  | 84.4 | [81.1, | 87.7] |  |  | 83.0 | [81.4, | 84.6] |  |  | 65.2 | [63.1, | 67.4] |  |  | 83.1 | [80.1, | 86.1] |  |
| Normal |  | 92.8 | [92.1, | 93.6] |  |  | 97.4 | [96.7, | 98.1] |  |  | 82.3 | [80.8, | 83.8] |  |  | 84.3 | [83.6, | 84.9] |  |  | 66.5 | [65.6, | 67.3] |  |  | 84.5 | [83.4, | 85.7] |  |
| Overweight |  | 93.6 | [92.5, | 94.7] |  |  | 97.9 | [97.0, | 98.8] |  |  | 82.2 | [79.9, | 84.4] |  |  | 85.6 | [84.6, | 86.5] |  |  | 67.8 | [66.7, | 69.0] |  |  | 85.0 | [83.4, | 86.6] |  |
| Obese |  | 91.1 | [88.5, | 93.8] |  |  | 96.0 | [93.8, | 98.1] |  |  | 82.9 | [79.1, | 86.8] |  |  | 86.5 | [84.9, | 88.2] |  |  | 66.0 | [63.8, | 68.2] |  |  | 81.9 | [78.6, | 85.1] |  |
|  |  |  |  |  |  |  |  |  |  |  |  |  |  |  |  |  |  |  |  |  |  |  |  |  |  |  |  |  |  |  |
| *Notes*. P is the p value from an F test of equal means across the respective adiposity categories | | | | | | | | | | | | | | | | | | | | | | | | | | | | | | |

| **Table S3. Adjusted differences in means of six HRQoL dimensions by BMI, Waist Circumference and Waist-Hip Ratio categories, and by sex** | | | | | | | | | | | | | | | | | | | | | | | | | | | | | | |
| --- | --- | --- | --- | --- | --- | --- | --- | --- | --- | --- | --- | --- | --- | --- | --- | --- | --- | --- | --- | --- | --- | --- | --- | --- | --- | --- | --- | --- | --- | --- |
|  |  | Physical Functioning | | | |  | Role Functioning | | | |  | Social Functioning | | | |  | Mental Health | | | |  | Health Perception | | | |  | Pain | | | |
|  |  | *Coeff. (95% CI)* | | *P* | |  | *Coeff. (95% CI)* | | *P* | |  | *Coeff. (95% CI)* | | *P* | |  | *Coeff. (95% CI)* | | *P* | |  | *Coeff. (95% CI)* | | *P* | |  | *Coeff. (95% CI)* | | *P* | |
| **MALES** |  |  |  | Indv. | Joint |  |  |  | Indv. | Joint |  |  |  | Indv. | Joint |  |  |  | Indv. | Joint |  |  |  | Indv. | Joint |  |  |  | Indv. | Joint |
| Body Mass Index |  |  |  |  |  |  |  |  |  |  |  |  |  |  |  |  |  |  |  |  |  |  |  |  |  |  |  |  |  |  |
| *Model 2* |  |  |  |  | 0.0085 |  |  |  |  | 0.2119 |  |  |  |  | 0.2362 |  |  |  |  | 0.7322 |  |  |  |  | 0.0035 |  |  |  |  | 0.5757 |
| Underweight |  | -4.93 | [-7.8,-2.1] | 0.001 |  |  | -0.77 | [-3.2,1.7] | 0.540 |  |  | -2.24 | [-7.6,3.1] | 0.414 |  |  | 0.31 | [-1.7,2.3] | 0.756 |  |  | -3.57 | [-6.4,-0.8] | 0.013 |  |  | 0.18 | [-3.3,3.7] | 0.922 |  |
| Acceptable risk |  |  | 0 |  |  |  |  | 0 |  |  |  |  | 0 |  |  |  |  | 0 |  |  |  |  | 0 |  |  |  |  | 0 |  |  |
| Increased risk |  | -0.01 | [-1.7,1.7] | 0.993 |  |  | 1.40 | [-0.0,2.8] | 0.052 |  |  | 0.53 | [-3.3,4.4] | 0.785 |  |  | 0.89 | [-0.7,2.5] | 0.267 |  |  | 1.91 | [-0.2,4.0] | 0.077 |  |  | 1.53 | [-1.3,4.3] | 0.287 |  |
| High risk |  | -0.19 | [-2.7,2.3] | 0.885 |  |  | 0.75 | [-1.1,2.6] | 0.429 |  |  | 4.92 | [-0.5,10.4] | 0.076 |  |  | 0.58 | [-1.8,2.9] | 0.623 |  |  | -0.37 | [-3.5,2.7] | 0.815 |  |  | -1.83 | [-6.7,3.0] | 0.461 |  |
|  |  |  |  |  |  |  |  |  |  |  |  |  |  |  |  |  |  |  |  |  |  |  |  |  |  |  |  |  |  |  |
| Waist |  |  |  |  |  |  |  |  |  |  |  |  |  |  |  |  |  |  |  |  |  |  |  |  |  |  |  |  |  |  |
| *Model 2* |  |  |  |  | 0.4394 |  |  |  |  | 0.0616 |  |  |  |  | 0.2676 |  |  |  |  | 0.2112 |  |  |  |  | 0.0275 |  |  |  |  | 0.7131 |
| Low |  |  | 0 |  |  |  |  | 0 |  |  |  |  | 0 |  |  |  |  | 0 |  |  |  |  | 0 |  |  |  |  | 0 |  |  |
| Borderline |  | 0.02 | [-1.7,1.8] | 0.980 |  |  | 1.58 | [0.3,2.9] | 0.020 |  |  | 1.18 | [-2.5,4.9] | 0.533 |  |  | 0.64 | [-0.9,2.1] | 0.400 |  |  | 2.39 | [0.4,4.4] | 0.018 |  |  | 0.61 | [-2.2,3.4] | 0.665 |  |
| High |  | -2.70 | [-6.9,1.5] | 0.204 |  |  | 0.04 | [-3.3,3.4] | 0.980 |  |  | 3.28 | [-2.7,9.3] | 0.284 |  |  | 2.16 | [-0.4,4.7] | 0.096 |  |  | -1.20 | [-4.9,2.5] | 0.520 |  |  | 1.99 | [-3.1,7.1] | 0.444 |  |
|  |  |  |  |  |  |  |  |  |  |  |  |  |  |  |  |  |  |  |  |  |  |  |  |  |  |  |  |  |  |  |
| Waist-hip ratio |  |  |  |  |  |  |  |  |  |  |  |  |  |  |  |  |  |  |  |  |  |  |  |  |  |  |  |  |  |  |
| *Model 2* |  |  |  |  | 0.0271 |  |  |  |  | 0.1866 |  |  |  |  | 0.1922 |  |  |  |  | 0.9349 |  |  |  |  | 0.7830 |  |  |  |  | 0.7488 |
| Low |  |  | 0 |  |  |  |  | 0 |  |  |  |  | 0 |  |  |  |  | 0 |  |  |  |  | 0 |  |  |  |  | 0 |  |  |
| Borderline |  | -1.63 | [-3.1,-0.1] | 0.035 |  |  | -1.09 | [-2.5,0.3] | 0.124 |  |  | -3.19 | [-6.6,0.3] | 0.069 |  |  | 0.22 | [-1.2,1.6] | 0.751 |  |  | 0.03 | [-1.7,1.8] | 0.971 |  |  | 0.79 | [-1.4,2.9] | 0.469 |  |
| High |  | -3.93 | [-7.6,-0.2] | 0.037 |  |  | -2.46 | [-5.9,1.0] | 0.158 |  |  | -0.90 | [-6.3,4.5] | 0.741 |  |  | -0.03 | [-2.3,2.2] | 0.978 |  |  | -1.16 | [-4.6,2.3] | 0.515 |  |  | 0.97 | [-3.2,5.2] | 0.649 |  |
| **FEMALES** |  |  | |  |  |  |  | |  |  |  |  | |  |  |  |  | |  |  |  |  | |  |  |  |  | |  |  |
| Body Mass Index |  |  |  |  |  |  |  |  |  |  |  |  |  |  |  |  |  |  |  |  |  |  |  |  |  |  |  |  |  |  |
| *Model 2* |  |  |  |  | 0.9001 |  |  |  |  | 0.9505 |  |  |  |  | 0.4748 |  |  |  |  | 0.4659 |  |  |  |  | 0.7482 |  |  |  |  | 0.9109 |
| Underweight |  | -0.04 | [-2.4,2.3] | 0.972 |  |  | 0.19 | [-1.8,2.2] | 0.847 |  |  | 1.75 | [-1.9,5.4] | 0.352 |  |  | -0.55 | [-2.4,1.3] | 0.553 |  |  | -0.18 | [-2.6,2.2] | 0.886 |  |  | -0.64 | [-3.9,2.6] | 0.697 |  |
| Acceptable risk |  |  | 0 |  |  |  |  | 0 |  |  |  |  | 0 |  |  |  |  | 0 |  |  |  |  | 0 |  |  |  |  | 0 |  |  |
| Increased risk |  | -0.25 | [-1.7,1.2] | 0.740 |  |  | 0.11 | [-1.2,1.4] | 0.866 |  |  | -0.84 | [-3.7,2.0] | 0.559 |  |  | -0.05 | [-1.2,1.1] | 0.932 |  |  | 0.73 | [-0.8,2.3] | 0.345 |  |  | -0.00 | [-2.1,2.1] | 0.998 |  |
| High risk |  | -0.70 | [-2.5,1.1] | 0.446 |  |  | -0.30 | [-1.8,1.2] | 0.694 |  |  | 1.47 | [-1.9,4.8] | 0.387 |  |  | 0.94 | [-0.5,2.4] | 0.199 |  |  | 0.58 | [-1.2,2.4] | 0.530 |  |  | -0.75 | [-3.4,1.9] | 0.572 |  |
|  |  |  |  |  |  |  |  |  |  |  |  |  |  |  |  |  |  |  |  |  |  |  |  |  |  |  |  |  |  |  |
| Waist |  |  |  |  |  |  |  |  |  |  |  |  |  |  |  |  |  |  |  |  |  |  |  |  |  |  |  |  |  |  |
| *Model 2* |  |  |  |  | 0.4234 |  |  |  |  | 0.5201 |  |  |  |  | 0.0369 |  |  |  |  | 0.9100 |  |  |  |  | 0.6741 |  |  |  |  | 0.9484 |
| Low |  |  | 0 |  |  |  |  | 0 |  |  |  |  | 0 |  |  |  |  | 0 |  |  |  |  | 0 |  |  |  |  | 0 |  |  |
| Borderline |  | -0.90 | [-2.6,0.8] | 0.284 |  |  | -0.31 | [-1.6,1.0] | 0.636 |  |  | -1.23 | [-4.0,1.6] | 0.389 |  |  | 0.12 | [-1.2,1.5] | 0.859 |  |  | -0.06 | [-1.7,1.6] | 0.944 |  |  | 0.35 | [-2.1,2.7] | 0.777 |  |
| High |  | -1.07 | [-2.7,0.6] | 0.198 |  |  | -0.73 | [-2.0,0.6] | 0.269 |  |  | -3.61 | [-6.4,-0.8] | 0.012 |  |  | 0.28 | [-1.0,1.6] | 0.678 |  |  | 0.56 | [-1.0,2.1] | 0.480 |  |  | 0.07 | [-2.2,2.4] | 0.953 |  |
|  |  |  |  |  |  |  |  |  |  |  |  |  |  |  |  |  |  |  |  |  |  |  |  |  |  |  |  |  |  |  |
| Waist-hip ratio |  |  |  |  |  |  |  |  |  |  |  |  |  |  |  |  |  |  |  |  |  |  |  |  |  |  |  |  |  |  |
| *Model 2* |  |  |  |  | 0.0965 |  |  |  |  | 0.1085 |  |  |  |  | 0.0006 |  |  |  |  | 0.1905 |  |  |  |  | 0.4078 |  |  |  |  | 0.9548 |
| Low |  |  | 0 |  |  |  |  | 0 |  |  |  |  | 0 |  |  |  |  | 0 |  |  |  |  | 0 |  |  |  |  | 0 |  |  |
| Borderline |  | 2.05 | [-1.6,5.7] | 0.272 |  |  | 1.29 | [-2.1,4.7] | 0.456 |  |  | 0.09 | [-5.0,5.2] | 0.972 |  |  | 1.01 | [-1.8,3.8] | 0.475 |  |  | 1.60 | [-1.7,4.9] | 0.338 |  |  | 0.49 | [-5.1,6.1] | 0.863 |  |
| High |  | 0.25 | [-3.1,3.6] | 0.885 |  |  | -0.01 | [-3.3,3.3] | 0.996 |  |  | -5.48 | [-10.0,-1.0] | 0.017 |  |  | -0.27 | [-2.7,2.2] | 0.829 |  |  | 0.55 | [-2.4,3.5] | 0.717 |  |  | 0.10 | [-4.9,5.1] | 0.968 |  |
| *Notes*. Multivariable ordinary least squares estimates adjusted for age categories, marital status, education, employment, urban/rural location, wealth quintile group, and smoking status (see Table 1). *P* indicates p value. *Indv*. gives p value from t-test of equality of means in category and reference category. *Joint* gives cate p value from F-test of equality of means across all categories. | | | | | | | | | | | | | | | | | | | | | | | | | | | | | | |

| **Table S4. Adjusted differences in means of six HRQoL dimensions by BMI, Waist Circumference and Waist-Hip Ratio categories, alternative specifications, Males** | | | | | | | | | | | | | | | | | | | | | | | | | | | | | | |
| --- | --- | --- | --- | --- | --- | --- | --- | --- | --- | --- | --- | --- | --- | --- | --- | --- | --- | --- | --- | --- | --- | --- | --- | --- | --- | --- | --- | --- | --- | --- |
|  |  | Physical Functioning | | | |  | Role Functioning | | | |  | Social Functioning | | | |  | Mental Health | | | |  | Health Perception | | | |  | Pain | | | |
|  |  | *Coeff. (95% CI)* | | *p* | |  | *Coeff. (95% CI)* | | *p* | |  | *Coeff. (95% CI)* | | *p* | |  | *Coeff. (95% CI)* | | *p* | |  | *Coeff. (95% CI)* | | *p* | |  | *Coeff. (95% CI)* | | *p* | |
| Body Mass Index |  |  |  | Indv. | Joint |  |  |  | Indv. | Joint |  |  |  | Indv. | Joint |  |  |  | Indv. | Joint |  |  |  | Indv. | Joint |  |  |  | Indv. | Joint |
| *Age adjusted* |  |  |  |  | 0.0027 |  |  |  |  | 0.2475 |  |  |  |  | 0.0541 |  |  |  |  | 0.1379 |  |  |  |  | 0.0014 |  |  |  |  | 0.6210 |
| Underweight |  | -5.58 | [-8.5,-2.6] | 0.000 |  |  | -1.22 | [-3.9,1.5] | 0.375 |  |  | -3.13 | [-8.5,2.3] | 0.256 |  |  | -0.51 | [-2.4,1.4] | 0.603 |  |  | -4.06 | [-6.8,-1.3] | 0.004 |  |  | -0.14 | [-3.7,3.4] | 0.937 |  |
| Acceptable risk |  |  | 0 |  |  |  |  | 0 |  |  |  |  | 0 |  |  |  |  | 0 |  |  |  |  | 0 |  |  |  |  | 0 |  |  |
| Increased risk |  | 0.04 | [-1.7,1.8] | 0.966 |  |  | 1.23 | [-0.1,2.6] | 0.080 |  |  | 1.39 | [-2.5,5.2] | 0.478 |  |  | 1.47 | [-0.2,3.1] | 0.079 |  |  | 1.57 | [-0.5,3.6] | 0.134 |  |  | 1.20 | [-1.5,3.9] | 0.378 |  |
| High risk |  | 0.10 | [-2.4,2.6] | 0.940 |  |  | 0.98 | [-0.9,2.8] | 0.305 |  |  | 5.79 | [0.6,11.0] | 0.030 |  |  | 1.65 | [-0.7,4.0] | 0.162 |  |  | -0.39 | [-3.5,2.7] | 0.807 |  |  | -2.01 | [-6.7,2.7] | 0.404 |  |
|  |  |  |  |  |  |  |  |  |  |  |  |  |  |  |  |  |  |  |  |  |  |  |  |  |  |  |  |  |  |  |
| *Fully adjusted* |  |  |  |  | 0.0087 |  |  |  |  | 0.2164 |  |  |  |  | 0.1610 |  |  |  |  | 0.7346 |  |  |  |  | 0.0050 |  |  |  |  | 0.6755 |
| Underweight |  | -4.70 | [-7.5,-1.9] | 0.001 |  |  | -0.60 | [-3.1,1.9] | 0.628 |  |  | -2.03 | [-7.5,3.4] | 0.465 |  |  | 0.30 | [-1.7,2.3] | 0.762 |  |  | -3.36 | [-6.1,-0.6] | 0.016 |  |  | 0.42 | [-3.1,3.9] | 0.813 |  |
| Acceptable risk |  |  | 0 |  |  |  |  | 0 |  |  |  |  | 0 |  |  |  |  | 0 |  |  |  |  | 0 |  |  |  |  | 0 |  |  |
| Increased risk |  | -0.04 | [-1.7,1.7] | 0.966 |  |  | 1.38 | [-0.0,2.8] | 0.054 |  |  | 0.50 | [-3.3,4.3] | 0.798 |  |  | 0.88 | [-0.7,2.5] | 0.271 |  |  | 1.88 | [-0.2,4.0] | 0.078 |  |  | 1.51 | [-1.3,4.3] | 0.294 |  |
| High risk |  | 0.50 | [-1.9,2.9] | 0.685 |  |  | 1.24 | [-0.6,3.1] | 0.183 |  |  | 5.60 | [0.2,11.0] | 0.041 |  |  | 0.62 | [-1.8,3.0] | 0.608 |  |  | 0.30 | [-2.7,3.3] | 0.848 |  |  | -1.17 | [-6.0,3.7] | 0.634 |  |
|  |  |  |  |  |  |  |  |  |  |  |  |  |  |  |  |  |  |  |  |  |  |  |  |  |  |  |  |  |  |  |
| Waist |  |  |  |  |  |  |  |  |  |  |  |  |  |  |  |  |  |  |  |  |  |  |  |  |  |  |  |  |  |  |
| *Age adjusted* |  |  |  |  | 0.5127 |  |  |  |  | 0.0661 |  |  |  |  | 0.2676 |  |  |  |  | 0.0177 |  |  |  |  | 0.0343 |  |  |  |  | 0.7636 |
| Low |  |  | 0 |  |  |  |  | 0 |  |  |  |  | 0 |  |  |  |  | 0 |  |  |  |  | 0 |  |  |  |  | 0 |  |  |
| Borderline |  | 0.36 | [-1.4,2.2] | 0.690 |  |  | 1.61 | [0.3,3.0] | 0.020 |  |  | 2.17 | [-1.6,5.9] | 0.260 |  |  | 1.55 | [-0.0,3.1] | 0.053 |  |  | 2.37 | [0.4,4.3] | 0.017 |  |  | 0.36 | [-2.3,3.0] | 0.792 |  |
| High |  | -2.21 | [-6.5,2.0] | 0.307 |  |  | 0.39 | [-3.1,3.8] | 0.825 |  |  | 4.27 | [-1.6,10.1] | 0.151 |  |  | 3.02 | [0.5,5.5] | 0.020 |  |  | -1.06 | [-4.8,2.7] | 0.581 |  |  | 1.81 | [-3.2,6.8] | 0.475 |  |
|  |  |  |  |  |  |  |  |  |  |  |  |  |  |  |  |  |  |  |  |  |  |  |  |  |  |  |  |  |  |  |
| *Fully adjusted* |  |  |  |  | 0.7176 |  |  |  |  | 0.0545 |  |  |  |  | 0.3078 |  |  |  |  | 0.1781 |  |  |  |  | 0.0325 |  |  |  |  | 0.4811 |
| Low |  |  | 0 |  |  |  |  | 0 |  |  |  |  | 0 |  |  |  |  | 0 |  |  |  |  | 0 |  |  |  |  | 0 |  |  |
| Borderline |  | 0.10 | [-1.7,1.9] | 0.908 |  |  | 1.64 | [0.3,3.0] | 0.016 |  |  | 1.28 | [-2.5,5.0] | 0.500 |  |  | 0.67 | [-0.8,2.2] | 0.386 |  |  | 2.47 | [0.6,4.4] | 0.012 |  |  | 0.67 | [-2.1,3.4] | 0.629 |  |
| High |  | -1.61 | [-5.6,2.4] | 0.432 |  |  | 0.82 | [-2.5,4.1] | 0.624 |  |  | 4.38 | [-1.3,10.0] | 0.127 |  |  | 2.27 | [-0.2,4.8] | 0.077 |  |  | -0.10 | [-3.8,3.6] | 0.958 |  |  | 3.07 | [-2.1,8.2] | 0.242 |  |
|  |  |  |  |  |  |  |  |  |  |  |  |  |  |  |  |  |  |  |  |  |  |  |  |  |  |  |  |  |  |  |
| Waist-hip ratio |  |  |  |  |  |  |  |  |  |  |  |  |  |  |  |  |  |  |  |  |  |  |  |  |  |  |  |  |  |  |
| *Age adjusted* |  |  |  |  | 0.0935 |  |  |  |  | 0.2632 |  |  |  |  | 0.3426 |  |  |  |  | 0.4540 |  |  |  |  | 0.8744 |  |  |  |  | 0.8073 |
| Low |  |  | 0 |  |  |  |  | 0 |  |  |  |  | 0 |  |  |  |  | 0 |  |  |  |  | 0 |  |  |  |  | 0 |  |  |
| Borderline |  | -1.27 | [-2.8,0.3] | 0.105 |  |  | -0.89 | [-2.2,0.4] | 0.189 |  |  | -2.46 | [-5.9,0.9] | 0.156 |  |  | 0.90 | [-0.5,2.3] | 0.212 |  |  | 0.11 | [-1.7,1.9] | 0.898 |  |  | 0.65 | [-1.5,2.8] | 0.547 |  |
| High |  | -3.27 | [-7.0,0.4] | 0.082 |  |  | -2.02 | [-5.4,1.3] | 0.237 |  |  | 0.29 | [-5.0,5.6] | 0.914 |  |  | 0.79 | [-1.5,3.1] | 0.502 |  |  | -0.79 | [-4.3,2.7] | 0.654 |  |  | 0.95 | [-3.2,5.1] | 0.655 |  |
|  |  |  |  |  |  |  |  |  |  |  |  |  |  |  |  |  |  |  |  |  |  |  |  |  |  |  |  |  |  |  |
| *Fully adjusted* |  |  |  |  | 0.0540 |  |  |  |  | 0.2582 |  |  |  |  | 0.2133 |  |  |  |  | 0.9371 |  |  |  |  | 0.8700 |  |  |  |  | 0.6411 |
| Low |  |  | 0 |  |  |  |  | 0 |  |  |  |  | 0 |  |  |  |  | 0 |  |  |  |  | 0 |  |  |  |  | 0 |  |  |
| Borderline |  | -1.49 | [-3.0,0.0] | 0.051 |  |  | -1.00 | [-2.4,0.4] | 0.160 |  |  | -3.06 | [-6.5,0.4] | 0.081 |  |  | 0.23 | [-1.1,1.6] | 0.740 |  |  | 0.17 | [-1.6,1.9] | 0.846 |  |  | 0.93 | [-1.2,3.1] | 0.392 |  |
| High |  | -3.47 | [-7.1,0.2] | 0.062 |  |  | -2.15 | [-5.5,1.2] | 0.211 |  |  | -0.45 | [-5.7,4.8] | 0.867 |  |  | 0.02 | [-2.3,2.3] | 0.986 |  |  | -0.68 | [-4.1,2.7] | 0.691 |  |  | 1.40 | [-2.8,5.6] | 0.514 |  |
| *Notes*. Multivariable ordinary least squares estimates. *Age adjusted* estimates adjusted for age categories only. *Fully adjusted* estimates adjusted for all covariates mentioned in notes to Table S3, plus history of chronic illnesses of participant and their immediate family. *P* indicates p value. *Indv*. gives p value from t-test of equality of means in category and reference category. *Joint* gives cate p value from F-test of equality of means across all categories. | | | | | | | | | | | | | | | | | | | | | | | | | | | | | | |

| **Table S5. Adjusted differences in means of six HRQoL dimensions by BMI, Waist Circumference and Waist-Hip Ratio categories, alternative specifications, Females** | | | | | | | | | | | | | | | | | | | | | | | | | | | | | | |
| --- | --- | --- | --- | --- | --- | --- | --- | --- | --- | --- | --- | --- | --- | --- | --- | --- | --- | --- | --- | --- | --- | --- | --- | --- | --- | --- | --- | --- | --- | --- |
|  |  | Physical Functioning | | | |  | Role Functioning | | | |  | Social Functioning | | | |  | Mental Health | | | |  | Health Perception | | | |  | Pain | | | |
|  |  | *Coeff. (95% CI)* | | *p* | |  | *Coeff. (95% CI)* | | *p* | |  | *Coeff. (95% CI)* | | *p* | |  | *Coeff. (95% CI)* | | *p* | |  | *Coeff. (95% CI)* | | *p* | |  | *Coeff. (95% CI)* | | *p* | |
| Body Mass Index |  |  |  | Indv. | Joint |  |  |  | Indv. | Joint |  |  |  | Indv. | Joint |  |  |  | Indv. | Joint |  |  |  | Indv. | Joint |  |  |  | Indv. | Joint |
| *Age adjusted* |  |  |  |  | 0.9025 |  |  |  |  | 0.9436 |  |  |  |  | 0.4539 |  |  |  |  | 0.0766 |  |  |  |  | 0.4663 |  |  |  |  | 0.8745 |
| Underweight |  | -0.16 | [-2.5,2.2] | 0.892 |  |  | 0.17 | [-1.8,2.2] | 0.867 |  |  | 2.00 | [-1.7,5.7] | 0.293 |  |  | -1.24 | [-3.1,0.6] | 0.196 |  |  | -0.51 | [-2.9,1.9] | 0.681 |  |  | -0.95 | [-4.3,2.3] | 0.570 |  |
| Acceptable risk |  |  | 0 |  |  |  |  | 0 |  |  |  |  | 0 |  |  |  |  | 0 |  |  |  |  | 0 |  |  |  |  | 0 |  |  |
| Increased risk |  | -0.20 | [-1.6,1.2] | 0.784 |  |  | 0.11 | [-1.2,1.4] | 0.867 |  |  | -0.86 | [-3.7,2.0] | 0.559 |  |  | 0.36 | [-0.8,1.5] | 0.549 |  |  | 0.93 | [-0.6,2.4] | 0.228 |  |  | 0.23 | [-1.8,2.3] | 0.828 |  |
| High risk |  | -0.68 | [-2.5,1.1] | 0.452 |  |  | -0.32 | [-1.7,1.1] | 0.663 |  |  | 1.28 | [-2.0,4.6] | 0.443 |  |  | 1.48 | [0.0,2.9] | 0.047 |  |  | 0.88 | [-0.9,2.7] | 0.335 |  |  | -0.47 | [-3.1,2.1] | 0.721 |  |
|  |  |  |  |  |  |  |  |  |  |  |  |  |  |  |  |  |  |  |  |  |  |  |  |  |  |  |  |  |  |  |
| *Fully adjusted* |  |  |  |  | 0.9196 |  |  |  |  | 0.8992 |  |  |  |  | 0.4923 |  |  |  |  | 0.4536 |  |  |  |  | 0.4714 |  |  |  |  | 0.8401 |
| Underweight |  | 0.04 | [-2.3,2.3] | 0.976 |  |  | 0.25 | [-1.7,2.2] | 0.805 |  |  | 1.83 | [-1.9,5.5] | 0.330 |  |  | -0.53 | [-2.4,1.3] | 0.569 |  |  | -0.17 | [-2.5,2.2] | 0.887 |  |  | -0.50 | [-3.7,2.7] | 0.758 |  |
| Acceptable risk |  |  | 0 |  |  |  |  | 0 |  |  |  |  | 0 |  |  |  |  | 0 |  |  |  |  | 0 |  |  |  |  | 0 |  |  |
| Increased risk |  | 0.01 | [-1.4,1.5] | 0.985 |  |  | 0.34 | [-0.9,1.6] | 0.601 |  |  | -0.65 | [-3.5,2.2] | 0.651 |  |  | 0.10 | [-1.1,1.3] | 0.866 |  |  | 1.12 | [-0.4,2.7] | 0.150 |  |  | 0.53 | [-1.5,2.6] | 0.613 |  |
| High risk |  | -0.56 | [-2.4,1.3] | 0.546 |  |  | -0.18 | [-1.6,1.3] | 0.809 |  |  | 1.59 | [-1.7,4.9] | 0.348 |  |  | 1.01 | [-0.4,2.5] | 0.167 |  |  | 0.72 | [-1.0,2.5] | 0.417 |  |  | -0.47 | [-3.1,2.1] | 0.724 |  |
|  |  |  |  |  |  |  |  |  |  |  |  |  |  |  |  |  |  |  |  |  |  |  |  |  |  |  |  |  |  |  |
| Waist |  |  |  |  |  |  |  |  |  |  |  |  |  |  |  |  |  |  |  |  |  |  |  |  |  |  |  |  |  |  |
| *Age adjusted* |  |  |  |  | 0.4686 |  |  |  |  | 0.4838 |  |  |  |  | 0.0488 |  |  |  |  | 0.2566 |  |  |  |  | 0.4009 |  |  |  |  | 0.8841 |
| Low |  |  | 0 |  |  |  |  | 0 |  |  |  |  | 0 |  |  |  |  | 0 |  |  |  |  | 0 |  |  |  |  | 0 |  |  |
| Borderline |  | -0.87 | [-2.5,0.8] | 0.299 |  |  | -0.32 | [-1.6,1.0] | 0.620 |  |  | -1.32 | [-4.1,1.5] | 0.352 |  |  | 0.60 | [-0.7,1.9] | 0.384 |  |  | 0.18 | [-1.5,1.8] | 0.831 |  |  | 0.57 | [-1.8,2.9] | 0.638 |  |
| High |  | -0.97 | [-2.6,0.6] | 0.231 |  |  | -0.74 | [-2.0,0.5] | 0.244 |  |  | -3.58 | [-6.5,-0.7] | 0.016 |  |  | 1.10 | [-0.2,2.4] | 0.102 |  |  | 0.99 | [-0.6,2.6] | 0.217 |  |  | 0.49 | [-1.8,2.8] | 0.674 |  |
|  |  |  |  |  |  |  |  |  |  |  |  |  |  |  |  |  |  |  |  |  |  |  |  |  |  |  |  |  |  |  |
| *Fully adjusted* |  |  |  |  | 0.5177 |  |  |  |  | 0.6812 |  |  |  |  | 0.0471 |  |  |  |  | 0.8256 |  |  |  |  | 0.4511 |  |  |  |  | 0.9083 |
| Low |  |  | 0 |  |  |  |  | 0 |  |  |  |  | 0 |  |  |  |  | 0 |  |  |  |  | 0 |  |  |  |  | 0 |  |  |
| Borderline |  | -0.84 | [-2.5,0.8] | 0.309 |  |  | -0.23 | [-1.5,1.0] | 0.718 |  |  | -1.22 | [-4.0,1.6] | 0.389 |  |  | 0.19 | [-1.2,1.5] | 0.782 |  |  | 0.21 | [-1.5,1.9] | 0.808 |  |  | 0.49 | [-1.9,2.8] | 0.682 |  |
| High |  | -0.89 | [-2.5,0.7] | 0.279 |  |  | -0.56 | [-1.9,0.7] | 0.398 |  |  | -3.50 | [-6.3,-0.7] | 0.016 |  |  | 0.40 | [-0.9,1.7] | 0.549 |  |  | 0.92 | [-0.6,2.5] | 0.245 |  |  | 0.44 | [-1.8,2.7] | 0.705 |  |
|  |  |  |  |  |  |  |  |  |  |  |  |  |  |  |  |  |  |  |  |  |  |  |  |  |  |  |  |  |  |  |
| Waist-hip ratio |  |  |  |  |  |  |  |  |  |  |  |  |  |  |  |  |  |  |  |  |  |  |  |  |  |  |  |  |  |  |
| *Age adjusted* |  |  |  |  | 0.1082 |  |  |  |  | 0.1057 |  |  |  |  | 0.0003 |  |  |  |  | 0.2389 |  |  |  |  | 0.4555 |  |  |  |  | 0.9421 |
| Low |  |  | 0 |  |  |  |  | 0 |  |  |  |  | 0 |  |  |  |  | 0 |  |  |  |  | 0 |  |  |  |  | 0 |  |  |
| Borderline |  | 1.69 | [-2.0,5.3] | 0.364 |  |  | 0.91 | [-2.5,4.3] | 0.600 |  |  | -0.43 | [-5.5,4.7] | 0.868 |  |  | 1.10 | [-1.7,3.9] | 0.439 |  |  | 1.52 | [-1.8,4.8] | 0.366 |  |  | 0.84 | [-4.8,6.5] | 0.770 |  |
| High |  | -0.10 | [-3.5,3.3] | 0.955 |  |  | -0.40 | [-3.7,2.9] | 0.810 |  |  | -6.19 | [-10.7,-1.7] | 0.007 |  |  | -0.10 | [-2.6,2.4] | 0.933 |  |  | 0.54 | [-2.5,3.6] | 0.727 |  |  | 0.46 | [-4.6,5.5] | 0.859 |  |
|  |  |  |  |  |  |  |  |  |  |  |  |  |  |  |  |  |  |  |  |  |  |  |  |  |  |  |  |  |  |  |
| *Fully adjusted* |  |  |  |  | 0.1019 |  |  |  |  | 0.1244 |  |  |  |  | 0.0006 |  |  |  |  | 0.2024 |  |  |  |  | 0.4494 |  |  |  |  | 0.9736 |
| Low |  |  | 0 |  |  |  |  | 0 |  |  |  |  | 0 |  |  |  |  | 0 |  |  |  |  | 0 |  |  |  |  | 0 |  |  |
| Borderline |  | 2.05 | [-1.5,5.7] | 0.263 |  |  | 1.27 | [-2.1,4.7] | 0.463 |  |  | 0.12 | [-4.9,5.2] | 0.964 |  |  | 0.98 | [-1.8,3.7] | 0.485 |  |  | 1.42 | [-1.8,4.6] | 0.379 |  |  | 0.47 | [-5.1,6.1] | 0.868 |  |
| High |  | 0.30 | [-3.0,3.6] | 0.859 |  |  | 0.02 | [-3.2,3.3] | 0.993 |  |  | -5.42 | [-9.9,-0.9] | 0.018 |  |  | -0.27 | [-2.7,2.2] | 0.828 |  |  | 0.46 | [-2.5,3.4] | 0.759 |  |  | 0.19 | [-4.8,5.2] | 0.940 |  |
| *Notes*. Multivariable ordinary least squares estimates. *Age adjusted* estimates adjusted for age categories only. *Fully adjusted* estimates adjusted for all covariates mentioned in notes to Table S3, plus history of chronic illnesses of participant and their immediate family. *P* indicates p value. *Indv*. gives p value from t-test of equality of means in category and reference category. *Joint* gives cate p value from F-test of equality of means across all categories. | | | | | | | | | | | | | | | | | | | | | | | | | | | | | | |

| Age adjusted |
| --- |
|  |
| Fully adjusted |
|  |
| Figure S1. Adjusted differences in means of health-related quality of life dimensions between categories of body mass index (BMI), waist circumference (WC), and waist-to-hip ratio (WHR) by sex  *Notes*. Multivariable ordinary least squares regression estimates. For each sex and HRQoL dimension, three regressions were estimated. One included BMI categories, another included WC categories, and the third included WHR categories. References categories are indicated by a dot at 0 with no whiskers that elsewhere indicate 95% CI. Age adjusted estimates adjusted for age categories only. Fully adjusted estimates adjust for all covariates in Table 1, with age entered as indicators of 5-year interval |
